# Evaluating an online training module on protecting children from secondhand smoke exposure: impact on knowledge, confidence and self-reported practice of health and social care professionals

**DOI:** 10.1186/s12889-015-2488-5

**Published:** 2015-11-16

**Authors:** Laura L. Jones, Andy McEwen

**Affiliations:** UK Centre for Tobacco and Alcohol Studies and Institute of Applied Health Research, Public Health Building, University of Birmingham, Edgbaston, Birmingham, B15 2TT UK; UK Centre for Tobacco and Alcohol Studies and Cancer Research UK Health Behaviour Research Centre, Epidemiology and Public Health, University College London, London, UK; National Centre for Smoking Cessation and Training, London, UK

**Keywords:** Secondhand smoke, Online training, Healthcare professionals, Knowledge, Self-reported practice, Very brief advice

## Abstract

**Background:**

Healthcare professionals report that a lack of training is the primary barrier to raising the issue of secondhand smoke (SHS). An open access online training module was therefore developed for those working with smoking families to deliver effective very brief advice on SHS. The current study aimed to evaluate the following: (1) does knowledge increase as a result of participating in the online training module, and (2) does the module impact on participant confidence and self-reported practice relating to SHS.

**Methods:**

Those accessing the module were invited to participate in an evaluation to assess participants’ knowledge about, and confidence in, delivering very brief advice on SHS. Change in knowledge was assessed via ten multiple choice questions and confidence was assessed by Likert scale responses to three statements. Data were collected across three time points: pre-training, post-training and after 3 months. Data were also collected at 3 months post module completion on self-reported changes in practice and key learning points.

**Results:**

Data at all three time points were available for 178 participants (~1 % of those who visited the module homepage over a 2 year period). Knowledge and confidence to deliver effective very brief advice for SHS significantly increased between the pre- and post-training assessments and was maintained at 3 months. Eighty-four percent self-reported that they perceived taking part in the training had led to positive changes in their clinical practice.

**Conclusions:**

There is potential for this module to be embedded within training programmes across health and social care professions, which may help to increase the knowledge and confidence of health and social care professionals to deliver very brief advice for SHS to smoking families. Future research needs to explore whether the smoking families who receive very brief advice for SHS are motivated to make changes to their home smoking behaviours and whether roll-out of this intervention would be cost-effective.

## Background

Exposure to secondhand smoke (SHS) has been causally linked with a number of childhood morbidities including respiratory infections, asthma and middle ear disease [1]. In addition, children who regularly see their parents smoking are more likely to become smokers themselves [2]. Globally, 40 % of children under the age of 14 years are regularly exposed to SHS and of the annual deaths linked to exposure, 28 % occur in children [3]. Whilst children’s exposure to SHS in England has declined markedly in recent years, 39 % over half (52 %) of children who live with at least one smoker are still regularly exposed to SHS at home [4]. The most effective way to reduce SHS exposure in children would be to encourage their caregivers to quit smoking altogether. However, for those caregivers who are unable or unwilling to quit, the next best option is to promote harm reduction strategies such as supporting them to make their homes smoke-free. It is important that complete smoking bans are introduced in households with children, rather than partial bans (such as smoking in one room), as stopping short of making the home completely smoke-free is unlikely to have a significant impact on children’s exposure [5].

Health and social care professionals (HCPs) are in a unique position to offer help and support to parents to make their homes smoke-free [6]. Practitioners working with smoking families unequivocally accept that children’s exposure to SHS is harmful to their health [7] and have reported a desire to engage in protecting children from the harms of SHS exposure [8]. However, they also indicate that although the majority of them felt able to raise the issue of SHS, they lacked knowledge and skills, as well as confidence, in providing practical support to caregivers to reduce SHS exposure in the home [8]. A lack of training has been identified as the primary barrier for HCPs to raising the issue of SHS with smoking families [8]. Confidence in raising the issue of SHS is higher for practitioners who have received formal training around SHS [8], indicating that training is particularly important and is beneficial to both the HCP and to the families receiving the support. Parents in the CEASE (Clinical Effort Against Secondhand Smoke Exposure) trial reported an increase in the rates of HCPs asking about parental smoking and smoke-free homes following paediatricians’ participation in online training in two low income clinics in the USA [9]. A further HCP brief advice training intervention rolled out by NHS Greater Glasgow and Clyde in 2009 showed that training was effective in helping to increase HCPs knowledge and confidence in raising the issue of SHS and to offer smoking families advice based on the 5As brief intervention framework [10]. Whilst shown to be effective, the training involved attending a 4 h face-to-face session, which may be impractical to roll out on a wider scale.

Three key SHS related training areas have been identified for HCPs who work with smoking families: (1) knowledge of SHS and its effects on children’s health, (2) how to raise the issue with caregivers in a non-confrontational way and (3) practical advice and tools on how to support caregivers to reduce SHS exposure in the home. The National Centre for Smoking Cessation and Training (NCSCT) developed a free online training module, funded by the English Department of Health. Very Brief Advice on Secondhand Smoke [11] involves establishing if smoking occurs in the home and car, advising on the benefits of going smoke-free and offering help and support. The aim of the current study was to evaluate this training module by addressing the following questions: (1) does knowledge increase as a result of participating in the online training module, (2) does participating in the online training module impact HCP confidence and practice relating to SHS and promoting smoke-free homes, and (3) are these changes maintained over time?

## Methods

### Development of the very brief advice intervention

The module being evaluated in this research is a 30-minute online training module developed by clinicians and academics to assist all health and social care professionals who work with children and families to raise the issue of SHS and promote action to reduce exposure in the home and car. The approach was adapted from a similar evidence-based training module [12] on delivering very brief advice on smoking for general practitioners and based upon a meta-analysis of brief smoking cessation interventions [13]. The NCSCT has developed a methodology for identifying evidence-based behaviour change techniques [14] and an analysis of the content of the module revealed the presence of 19 (of 71) such techniques, as described elsewhere [11]. The training is based around a promotional film, short film clips demonstrating possible interactions with families, plus facts, figures and strategies to help build knowledge and skills in this area. The module provides accessible information on four key themes: (1) the harms caused by SHS, (2) why it is important to raise the issue, (3) how to ask, advise and act, and (4) encouraging and supporting behaviour change. A short assessment (multiple-choice questions (MCQs)) forms part of the training and a certificate is issued following successful completion.

### Evaluation of the training module

All participants who accessed the very brief advice for SHS training homepage between the launch in April 2012 and March 2014 were invited to participate in an evaluation of the module prior to undertaking the training. This included information on what participating in the evaluation would involve and provided participants with a choice to opt in to the research and so written informed consent was not obtained.

Evaluation participants were not offered any type of incentive to take part. Those opting to participate were initially asked about: gender, age, profession, how long they have been in their current profession, and how long they have been in their current post. Following this, they were asked to rate how confident they felt in raising SHS with smoking families via the following three questions using a five point Likert scale: (1) strongly agree through to (5) strongly disagree:

1. I am confident in raising the issue of secondhand smoke exposure with my clients
2. I am confident in raising the idea of smoke-free homes and cars with my clients
3. I am confident in my ability to offer practical help and support to my clients around making their home and car smoke-free

Prior to entering the training module, participants completed a knowledge test of ten MCQs multiple-choice questions. The knowledge test covered the five main components of delivering very brief advice:

1. Who should receive very brief advice on secondhand smoke
2. Dangers of secondhand smoke
3. Clients reactions to very brief advice on secondhand smoke
4. Ask/Advise element of very brief advice for secondhand smoke
5. Advise/Act element of very brief advice for secondhand smoke

We developed three similar MCQs questions for each section and then the training software was used to randomly draw two questions per section to generate a knowledge test comprising 10 MCQs for each participant. This process was used for the tests at all three time points and was done to minimise the risk of the test answers being shared amongst participants. At the end of the training a further 10 MCQs were completed and they were asked the same three confidence questions. Participants were contacted by email 3 months after accessing the training and asked to complete the same three confidence questions, 10 MCQs and a further question exploring whether their practice had been influenced by participating in the training:

1. Has the very brief advice for SHS training module resulted in you improving the way you provide support to smoking parents and carers? (yes/no/unsure)

### Analysis

An *a priori* pass mark of 80 % (correctly answering eight out of ten randomly selected questions) was set for the knowledge multiple-choice assessments at each time point. Binary variables (all answers correct for that knowledge component vs. one or more incorrect answers for that knowledge component) were computed for each of the five topics covered within the pool of 15 questions to allow an exploration of knowledge of the individual components covered in the very brief advice for SHS training module and if this varied over time. For the three questions on confidence, the combined percentage of participants who responded ‘agree’ or ‘strongly agree’ was computed. Descriptive statistics were used to describe the sample and McNemar tests used to examine differences between time points (pre vs. post, pre vs. follow up and post vs. follow up) for both knowledge and confidence. The level of statistical significance was set to *p* < 0.05 for all analyses.

### Ethical considerations

Formal ethics approval was not required because the study was deemed to be a service evaluation.

## Results

### Evaluation sample

Between the launch of the training module in April 2012 and March 2014, 20,578 participants accessed the very brief advice for SHS training homepage. Of these, 1,296 (6.3 %) participated in the pre-training evaluation, 904 (4.4 %) in the post-training evaluation and 431 (2.1 %) in the 3 month evaluation. In total, complete datasets across the three time points were available for 178 participants (0.9 %). Table 1 provides a summary of the demographic characteristics of the evaluation sample. The vast majority of participants were female, over the age of 50 years and working either as a stop smoking practitioner or other health and social care professional (e.g. health trainers, health improvement/promotion practitioners and community health workers).

**Table 1:** Demographic characteristics of Very Brief Advice for Secondhand Smoke evaluation sample (*n* = 178)

		% (n)
Gender	Female	84 (149)
	Male	16 (29)
Age	<29 years	11 (20)
	30–39 years	17 (30)
	40–49 years	34 (60)
	>50 years	38 (68)
Profession	GP/Hospital doctor	2 (3)
	Nurse, midwife, health visitor	15 (27)
	Other	13 (23)
	Other Health/Social Care Professional	27 (49)
	Pharmacist	6 (11)
	Stop smoking practitioner	37 (65)

### Knowledge

Overall, the percentage of participants displaying the knowledge required for effective delivery of very brief for SHS (at least 8/10 in the MCQ assessment) improved between the pre- and post-training assessments and was maintained at 3 months compared to the pre-training score (Table 2). When knowledge required for effective delivery of very brief advice of SHS was analysed by the five key components being tested; most participants had good knowledge of who should receive very brief advice for SHS prior to taking part in the training and this awareness was maintained over the 3 month follow up period. Scores for the four other areas of very brief advice for SHS increased between pre- and post-training and were maintained over the 3 month follow up period, with the exception of the very brief advice Advise/Act components which increased pre- to post-training, but decreased at 3 months. Participants had relatively low knowledge of the dangers of SHS prior to the training, with only three quarters of the sample answering two out of two questions correctly; this increased following the training and was maintained over time. Less than one third of the participants were able to answer two out of two questions correctly on the Ask/Advise elements of very brief advice prior to the training; this increased following the training but decreased at 3 months, although this was still significantly higher than the pre-training score.

**Table 2:** Changes in knowledge to deliver effective very brief advice on secondhand smoke and smoke-free homes/SFH homes prior to and after training

Knowledge	Pre-training % (n)	Post-training % (n)	Three months follow up % (n)	p value(pre vs. post)	p value (pre vs. follow up)	p value (post vs. follow up)
Overall score (answered eight of ten questions correctly)	85 (151)	97 (172)	94 (168)	<0.001	0.001	0.388
Knowledge split by area of VBA for SHS tested						
Who should receive very brief advice for SHS? (answered two out of two questions correctly)	95 (168)	97 (172)	96 (170)	0.424	0.791	0.804
Dangers of SHS (answered two out of two questions correctly)	74 (131)	88 (157)	84 (150)	<0.001	0.007	0.324
Client reactions to very brief advice for SHS (answered three of three questions correctly)	83 (147)	92 (164)	89 (158)	<0.001	0.035	0.18
Ask/Advice elements of very brief advice for SHS (answered two out of two questions correctly)	29 (51)	81 (144)	64 (114)	<0.001	<0.001	<0.001
Advise/Act elements of very brief advice for SHS (answered one out of one question correctly)	77 (137)	93 (166)	83 (147)	<0.001	0.144	0.002

### Confidence

The number of subjects displaying confidence in all three areas assessed increased between pre- and post-training: raising the issue of secondhand smoke; raising the idea of smoke-free homes and cars; and offering practical help and support around smoke-free homes and cars. This increase in confidence was maintained at 3 months for all three areas (Table 3).

**Table 3:** Changes in confidence to deliver effective very brief advice on secondhand smoke (SHS) and smoke-free homes prior to and after training

Confidence	Pre-training % agree or strongly agree (n)	Post-training % agree or strongly agree (n)	Three months follow up % agree or strongly agree (n)	p value (pre vs. post)	p value (pre vs. follow up)	p value (post vs. follow up)
Confidence to raise the issue of SHS	85 (152)	96 (170)	97 (173)	<0.001	<0.001	0.508
Confidence to raise to the idea of smoke-free homes and cars	87 (154)	97 (172)	96 (170)	<0.001	0.002	0.754
Confidence to offer practical help and support around smoke-free homes and cars	80 (142)	96 (171)	96 (170)	<0.001	<0.001	1.000

### Changes to practice

Eighty-four percent (*n* = 147) of the evaluation sample self-reported at the 3 month follow up that they perceived taking part in the online training module had led to positive changes in their practice when working with smoking families. With only a minority reporting that taking part in the training had not influenced their practice (6 %, *n* = 11) or that they were unsure (10 %, *n* = 18).

## Discussion

Participation in the open access online training module significantly improved knowledge and increased confidence to deliver effective very brief advice for SHS for health and social-care professionals who work with smoking families, and these increases were maintained over the 3 month follow up period. Overall knowledge on how to deliver effective very brief advice for SHS was high for participants prior to enrolment in the training module. However, when knowledge was explored by the different components tested, it was apparent that whilst participants had good awareness of who should receive very brief advice for SHS and typical clients reactions to brief advice, they lacked knowledge about the dangers of SHS and found it difficult to understand what topics should be covered in a conversation around SHS and smoke-free homes with smoking families. This lack of knowledge was also reflected in reduced confidence in raising the issue of SHS and, in particular, in providing practical help and support around making changes to home smoking behaviour. These findings are in line with a previous survey of HCPs in Scotland who reported that whilst they felt able to raise the issue of SHS, they lacked knowledge and skills, as well as confidence, in providing practical support to caregivers to reduce SHS exposure in the home [8].

This novel training is one of very few, if not the only, freely accessible online modules available in the UK (and wider) to support professionals working with smoking families to deliver effective very brief advice for SHS. There is potential given that only a very small minority (~1 %) of the large number of people who visited the module homepage during the study period opted to participate in the evaluation, that the sample is biased towards more knowledgeable and motivated HCPs. In addition, it is important to note that visiting the module homepage may not reflect the actual number of people who completed the training thus the response rate reported may be an underestimate of the true response rate.

The low response rate and small sample size are clear limitations of the current study. However, it was evident that participants within this small sample did lack knowledge and confidence in providing very brief advice for SHS prior to taking the module. A further limitation of the study design was that the training software (which is now obsolete) did not allow us to report on whether any participants received the same questions at all three time points, nor on the relative difficulty of individual MCQs. Although it was not possible to compare participants with a control group of HCPs who did not participate in the training, it is reasonable to assume that the improvements in participants’ knowledge and confidence, and the fact these changes were maintained over time, were due to participation in the online training module. It is also important to consider that the assessment of knowledge and confidence used in the current study may not in reality reflect a family’s ability to make changes to their home smoking, or the likely success of any changes made in protecting children from the harms of SHS. Therefore, it would be helpful if future studies explored with families, who have received very brief advice for SHS from a HCP trained via this module, if they were able to initiate and maintain a smoke-free home. In addition, it would be helpful to explore if the cost of 30 min of HCPs time to complete the module is offset by a reduction in costs and burden of disease caused by exposure to SHS. Training in smoking cessation is sub-optimal in UK medical schools [15], in schools of nursing [16] and there is no evidence of training in SHS interventions. This study is the first of its kind to evaluate a SHS online training module and it in part answers recent calls for tobacco control training to be evidence-based and rigorously evaluated [17]. In fact this study, by following up participants at 3 months and enquiring about changes to clinical practice, goes beyond evaluation which just focuses on training content and delivery.

## Conclusions

For the sample of HCPs who took part in the evaluation, knowledge and confidence to deliver effective very brief advice for SHS significantly improved following participation in the online training module and these increases were maintained for at least 3 months. Participation also led to self-reported positive changes in practice when working with smoking families. This novel training takes only 30 min to complete and is freely accessible to any health and social-care professional with access to the internet. There is potential for this module to be embedded within training programmes across health and social care professions, which may help to increase the knowledge and confidence of HCPs to deliver very brief advice for SHS to smoking families. Future research needs to explore whether the smoking families who receive very brief advice for SHS are motivated to make changes to their home smoking behaviours and whether roll-out of this intervention would be cost-effective.

## Abbreviations

HCP/s: Health and social care professional/s
SHS: Secondhand smoke

## References

[1] Royal College of Physicians (2010). Passive smoking and children. A report by the Tobacco Advisory Group.

[2] Leonardi-Bee J, Jere ML, Britton J (2011). Exposure to parental and sibling smoking and the risk of smoking uptake in childhood and adolescence: a systematic review and meta-analysis. Thorax.

[3] Oberg M, Jaakkola MS, Woodward A, Peruga A, Pruss-Ustun A (2011). Worldwide burden of disease from exposure to second-hand smoke: a retrospective analysis of data from 192 countries. Lancet.

[4] Jarvis MJ, Feyerabend C (2015). Recent trends in children's exposure to second-hand smoke in England: cotinine evidence from the Health Survey for England. Addiction.

[5] Wakefield M, Banham D, Martin J, Ruffin R, McCaul K, Badcock N (2000). Restrictions on smoking at home and urinary cotinine levels among children with asthma. Am J Prev Med.

[6] Ritchie D, Amos A, Phillips R, Cunningham-Burley S, Martin C (2009). Action to achieve smoke-free homes: an exploration of experts’ views. BMC Public Health.

[7] Ritchie DD, Amos A, Shaw A, O’Donnell R, Semple S, Turner S, Martin C. How do policy advisors and practitioners prioritise the protection of children from secondhand smoke exposure in a country with advanced tobacco control policy?. Tob Control. 2013. doi:10.1136/tobaccocontrol-2012-050936. 10.1136/tobaccocontrol-2012-050936 23956059

[8] Reducing Families’ Exposure to Second-Smoke in the Home: Survey of Professionals working with Families and Children. 2010. [http://www.ashscotland.org.uk/media/3779/Practitioners%20Baseline%20Survey%20%20ASH%20Scotland%20%202010.pdf] Accessed 16 Oct 2015.

[9] Hipple B, Nabi-Burza E, Hall N, Regan S, Winickoff JP (2013). Distance-based training in two community health centers to address tobacco smoke exposure of children. BMC Pediat.

[10] Gordon J, & Associates. Evaluation of Training to Reduce Children’s Exposure to Second Hand Smoke. In*.*: A Report Commissioned by NHS Greater Glasgow and Clyde. 2010.

[11] Secondhand Smoke training module [http://www.ncsct.co.uk/publication_secondhand-smoke-training-module.php] Accessed 16 Oct 2015.

[12] Very Brief Advice Training Module [http://www.ncsct.co.uk/publication_very-brief-advice.php] Accessed 16 Oct 2015.

[13] Aveyard P, Begh R, Parsons A, West R (2012). Brief opportunistic smoking cessation interventions: a systematic review and meta-analysis to compare advice to quit and offer of assistance. Addiction.

[14] Michie S, Churchill S, West R (2011). Identifying evidence-based competences required to deliver behavioural support for smoking cessation. Ann Behav Med.

[15] Raupach T, Al-Harbi G, McNeill A, Bobak A, McEwen A (2015). Smoking cessation education and training in U.K. medical schools: a national survey. Nicotine Tob Res.

[16] Richards B, McNeill A, Croghan E, Percival J, Ritchie D, McEwen A (2014). Smoking cessation education and training in UK nursing schools: A national survey. J Nurs Educ Pract.

[17] Selby P, Goncharenko K, Barker M, Fahim M, Timothy V, Dragonetti R, Kemper K, Herie M, Hays JT (2015). Review and evaluation of online tobacco dependence treatment training programs for health care practitioners. J Med Internet Res.

